# Hybrid Electrospun Fibers for Rapid Delivery of *Lactobacillus paragasseri* K7 and Lactoferrin as Live Biotherapeutics and Postbiotics

**DOI:** 10.1021/acsomega.5c07130

**Published:** 2026-01-23

**Authors:** Marjana Simonič, Bojana Bogovič Matijašić, Petra Mohar Lorbeg, Zdenka Peršin Fratnik, Lidija Fras Zemljič

**Affiliations:** † Laboratory of Water Physics and Membrane Processes, Faculty of Chemistry and Chemical Engineering, 54765 University of Maribor, 20000 Maribor, Slovenia; ‡ 37663 University of Ljubljana, Biotechnical Faculty, Department of Animal Science, Institute of Dairy Science and Probiotics, 1230 Domžale, Slovenia; § Faculty of Medicine, 54765 University of Maribor, 2000 Maribor, Slovenia; ∥ Laboratory for Characterization and Processing of Polymers, Institute of Engineering Materials and Design, Faculty of Mechanical Engineering, 54765 University of Maribor, 2000 Maribor, Slovenia

## Abstract

This study explores the development of electrospun nanofibrous materials as delivery systems for the probiotic strain *Lactobacillus paragasseri* K7 (LK7) and the bioactive glycoprotein lactoferrin (LF) with applications targeting vaginal health. Electrospinning was used to encapsulate LK7 and LF into poly­(ethylene oxide) (PEO)-based nanofibers supported on polypropylene fabric. Three formulations—PEO/LF, PEO/lactobacilli (LB) (with LK7), and PEO/LF/LB—were characterized for their physicochemical properties, fiber morphology (SEM), chemical composition (FTIR, XPS), and antioxidant activity (2,2′-azino-bis­(3-ethylbenz-thiazoline-6-sulfonic acid) (ABTS) assay). SEM analysis confirmed successful nanofiber formation, though LK7 remained on the fiber surface due to its size. FTIR and XPS analyses verified the incorporation of functional groups and elements associated with LF and LK7. The antioxidant assays showed that both LF and LK7 exhibited strong radical scavenging activity in formulations and it decreased slightly after electrospinning. Among the electrospun samples, the PEO/LF/LB formulation demonstrated the highest antioxidant potential. The viability and release studies revealed that 0.38–0.45% of LK7 survived during the electrospinning process and that the bacterial cells were released rapidly within 1 min of PBS exposure. Storage at 8 or 20 °C under 65% humidity reduced the viability (cfu) further, likely due to a transition to a viable but nonculturable (VBNC) state. Despite the low survival rates, the immediate release profile and antimicrobial potential of the materials support their suitability for short-term therapeutic applications such as vaginal tampons or wound dressings. This study highlights the potential of nozzle-free electrospinning for developing delivery systems for live biotherapeutics and postbiotics and suggests future work to optimize viability or expand into postbiotic applications.

## Graphical abstract

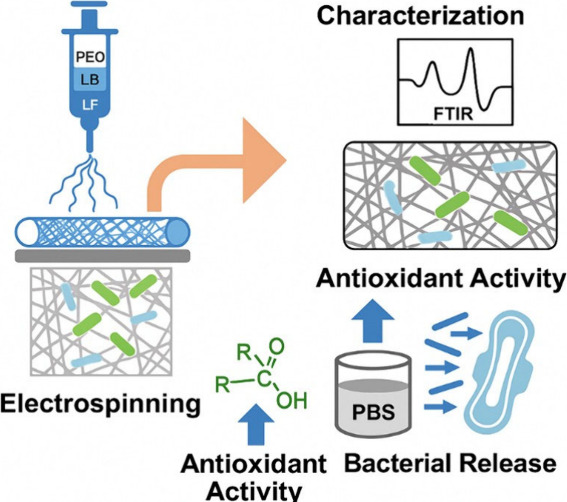

## 1 Introduction

Lactobacilli are strong candidates for use in live biotherapeutic products (LBPs) due to their well-established safety profile and ability to modulate immune responses, inhibit pathogens, and support mucosal barrier function. These beneficial effects have been demonstrated not only in the gastrointestinal tract but also in respiratory and urogenital applications.^1,2^ In contrast to conventional probiotics—marketed primarily as dietary supplements with general health claims—LBPs are regulated as pharmaceuticals and must demonstrate therapeutic efficacy for specific clinical indications through controlled trials.^3^

A growing body of research has highlighted the potential of combining lactobacilli with bioactive compounds that possess prebiotic properties to enhance their functionality and viability.^4^ One such compound is lactoferrin (LF), an iron-binding glycoprotein present naturally in various secretory fluids, including milk, saliva, and vaginal secretions. LF not only is antimicrobial and immunomodulatory but also exhibits prebiotic effects by promoting the growth of beneficial bacteria.^5^ The synergy between lactobacilli (LB) and lactoferrin is particularly promising: while lactobacilli suppress pathogen proliferation and modulate mucosal immunity, lactoferrin can block microbial adhesion to host epithelial cells and amplify antimicrobial defense mechanisms.^5,6^ Together, they can prevent the colonization of pathogens and protect still healthy cells from infection.

This cooperative effect is especially relevant in the female reproductive tract, where bacterial and fungal infections—such as bacterial vaginosis, candidiasis, or *Chlamydia trachomatis* infections—are often associated with dysbiosis of the vaginal microbiota. Several studies have demonstrated that LF and LB, particularly in combination, can restore vaginal health and reduce inflammation, serving as either monotherapy or adjunctive treatment alongside conventional antimicrobial agents.^7,8^ Their combined activity has also been shown to block the early stages of *C. trachomatis* infection in cervical epithelial cells, thereby reducing the risk of complications such as infertility, chronic pelvic pain, or dysplasia.

In terms of application, both oral and local (e.g., vaginal) routes are possible, but local delivery systems such as gels, tampons, pads, or suppositories are more effective for acute infections. They allow a high concentration of viable bacteria to reach the mucosal surface rapidly, promoting immediate colonization and therapeutic action.^8^

In recent years, electrospinning has emerged as a promising platform for the encapsulation and delivery of live biotherapeutic agents. This technique enables the formation of nanofibrous matrices under mild (nonthermal) conditions, preserving bacterial viability and ensuring high encapsulation efficiency.^9^ Various studies have shown that the use of protective excipients such as lactose, mannitol, or skim milk powder during electrospinning with poly­(ethylene oxide) (PEO) enhances bacterial survival, often exceeding 80%.^10^ Furthermore, the addition of sodium alginate or other polysaccharides can allow for controlled and extended release of probiotics for up to 24 h.^11^ Electrospun materials are already being investigated for drug delivery, wound healing, and mucosal therapies.^12^ The fabricated zein/sakacin nanofibers were shown to be promising nanostructures in the active packaging of ready-to-eat products with no environmental repercussions.^13^ Core–sheath fibers have emerged as versatile and structurally efficient approaches as they provide a holistic, structurally embedded functionality in contrast to surface coated fibers.^14,15^ However, the need for precise control over processing parameters can make production complicated.^16^ As sustainability has become a key priority in fiber manufacturing, the pressurized spinning method has evolved toward a more energy-efficient and sustainable approach.^17,18^ Despite being a highly efficient spinning method, its major disadvantage is the production of less uniform nanostructures.

LF exhibits antimicrobial action and immunomodulatory effects, thus contributing to the innate immune system of mammals.^19^ Lactoferrin-loaded polycaprolactone matrices were found as a promising vaginal delivery system for controlled release in the treatment of infections.^20^ The notable increased cellular uptake of carboplatin-loaded lactoferrin nanoparticles has been documented as compared to their standard counterparts.^21^

The most common probiotics/live biotherapeutics are representatives of the *Lactobacillaceae* family, characterized by the production of organic acids and bacteriocins, which are also the main cause of their antibacterial activity.^22^ They are applied in the field of the food industry in fermented products and also increasingly in the field of medical applications. Lactobacilli can act as biotherapeutic agents in the treatment of urinary tract infections, suppression of genital inflammatory processes, and cholesterol reduction.^23^ Lactobacilli as live biotherapeutics present no drawbacks for healthy people and no warning of side effects.^24^ In addition to their contribution to the production of many fermented foods, lactobacilli are also important components of human gut microbiota. In general, the members of the species *Lactobacillus (L.) paragasseri* are commensal bacteria that inhabit humans in the oral cavity, vagina, or digestive tract.^25^

Despite the growing body of evidence supporting the independent antimicrobial and immunomodulatory roles of both lactobacilli and lactoferrin, their simultaneous encapsulation in a single, stable delivery system remains underexplored—particularly in the context of vaginal health. Existing formulations often rely on either freeze-dried bacterial forms or separate application strategies, which may limit the colonization efficiency or the synergistic interaction at the target site. Electrospinning offers a unique platform to codeliver these bioactives in a nonthermal, high-efficiency process that preserves viability and biofunctionality. By leveraging nanofiber morphology, surface area, and release dynamics, electrospun materials could, thus, bridge the gap between biological efficacy and technological feasibility. A practical strategy for the fabrication of biobased nanocarriers was presented by Dai et al.^26^ The addition of sodium dodeyl sulfate in gelatin emulsion was crucial for production of uniform nanofibers.

This study therefore aims to develop and characterize electrospun nanofibers based on poly­(ethylene oxide) (PEO) that deliver *Lactobacillus paragasseri* K7 (a strain producing gassericins K7A and K7B) and lactoferrin simultaneously. This strain, isolated from infant feces, is known for its antimicrobial activity and probiotic robustness.^27,28^ The research focuses on evaluating the morphology, physicochemical properties, antioxidant activity, and microbiological performance of the developed materials, including the bacterial viability and release kinetics. The ultimate goal is to explore their potential for topical intravaginal application—as tampons or wound dressings—that could maintain or restore a healthy vaginal microbiota during periods of infection, inflammation, or bleeding.^29,30^

## 2 Materials and Methods

### 2.1 Chemicals

The spray dried Lactoferrin (LF, 213545 SLFLF) was kindly provided by the Arhel company (Komenda, Slovenija).^31^

An ABTS reagent (2,2′-azino-bis­(3-ethylbenz-thiazoline-6-sulfonic acid) (Sigma-Aldrich, Germany)) was used to prepare a solution for determining the antioxidant activity.

MRS Broth (Merck, Germany) was used for the cultivation of the lactobacilli. Rogosa Agar (Merck, Germany) was used for enumeration of lactobacilli by standard plate counting.

The phosphate buffer (PBS, pH 7.4) contained NaCl (16.0 g), KCl (0.4 g), NaHPO_4_ (1.8 g), and KH_2_PO_4_ (0.49 g).

Poly­(ethylene oxide) (M = 600 000 g/mol, Across Organics, Geel, Belgium) was dispersed in Milli-Q water to prepare a 5% (w/w) PEO solution. It was stirred for 24 h using a propeller stirrer.

The *Lactobacillus paragasseri* K7 (ZIM 105, Zbirka industrijskih mikroorganizmov–WFCC #810, Ljubljana, Slovenia; CCM 7710, Czech Collection of Microorganisms, Brno, Czech Republic) was propagated from frozen stocks by incubation in MRS broth.

The abbreviations of the electrospinning formulations (s = solution) and electrospun material are listed in Table 1.

**Table 1:** Abbreviations of Electrospinning Formulations and Electrospun Material^a^

s-PP	Polypropylene
s-PEO	5% Poly(ethylene oxide) solution (PEO)
p-LF	Lactoferrin
s-LB	*L. paragasseri* K7
s-PEO/LF	Formulation 5% solution PEO and lactoferrin
s-PEO/LB	Formulation 5% solution PEO and *L. paragasseri* K7
s-PEO/LF/LB	Formulation 5% solution PEO, lactoferrin and *L. paragasseri* K7
PP	Electrospun polypropylene
PEO	Electrospun PEO
PEO/LF	Electrospun PEO and lactoferrin
PEO/LB	Electrospun PEO and *L. paragasseri* K7
PEO/LF/LB	Electrospun PEO, lactoferrin, and *L. paragasseri* K7

a s = suspension.

### 2.2 Methods

#### 2.2.1 Preparing the Electrospinning Formulations

The *L. paragasseri* K7 overnight culture was inoculated (1% v/v) in 500 mL of MRS broth (Fluka Analytical, UK). Incubation of the culture took place overnight at 37 °C. The biomass was collected by centrifugation (10 min, 8000*g*), washed with buffer PBS, vortexed, and centrifuged (5 min, 3300*g*). The pellet containing K7 cells was resuspended in 9 mL of Ringer solution (s-LB) and transferred to the electrospinning solutions.

The electrospinning formulations were prepared by mixing 30 mL of PEO solution (s-PEO) with (i) 0.3 g of lactofferin (s-PEO/LF), (ii) a concentrated lactobacilli suspension containing approximately 1.3·10^10^ cfu/mL (s-PEO/LB), or (iii) lactoferrin (0.3 g) and lactobacilli (1.3·10^10^ cfu/mL). The suspensions were prepared under continuous stirring at room temperature until completely homogeneous. All the used polymer formulations were mixed with a propeller mixer for 15 min before characterization and electrospinning.

#### 2.2.2 Characterization of the Electrospinning Formulations

The efficiency of nanofiber formation depends on the polymer solution properties used in electrospinning. The solution samples, as listed in Table 1, were characterized prior to the electrospinning process. Accordingly, the conductivity, pH, viscosity, and surface tension were determined using a HPC 227K conductivity meter (Mettler Toledo, Columbus, OH, United States), a Seven Compact pH meter (Metler Toledo, Columbus, OH, United States), rotation, a capillary rheometer (FUNGILAB, Barcelona, Spain), and a K 12 Tensiometer (Krüss GmbH, Hamburg, Germany).

#### 2.2.3 Formation of Nanofibers by Electrospinning

Nozzle-free centrifugal electrospinning was carried out using a NanoSpider NS LAB 500 (Elmarco, Liberec, Czech Republic) via the needle-free technique. A bathtub filled with the polymer solution (30 mL) containing the spinning electrode was placed into the apparatus. The polypropylene material (30 cm × 40 cm), as standard basic material (Pegatex S nonwoven, kindly supplied by PEGAS NONWOVENS s.r.o., Znojmo, Czech Republic in the form of 100% polypropylene (PP) fiber mesh), was placed on the upper rounded collecting electrode and used as material for collecting the formed nanofibers.

Conductivity (κ), pH, surface tension (γ), and viscosity (η) were determined to define the optimal concentration and volume ratio for smooth electrospinning. The pH was determined using a Seven Compact Mattler Toledo pH meter (Columbus, USA); conductivity was determined using a HPC 227K Metler Toledo conductivity meter (Columbus, USA). The surface tension was determined by a K12 Krüss Tensiometer (Hamburg, Germany), and the viscosity was determined by rotation and a capillary rheometer FUNGILAB (Barcelona, Spain).

Besides the physical properties of the polymer solutions, the environmental and technological parameters also influence the formation of nanofibers. Hence, optimization of the electrospinning procedure followed, varying the processing parameters, such as voltage (*U*) and distance between the electrodes (*d*), and optimization was also performed for the environmental conditions (temperature (*T*) and relative humidity (RH)).

#### 2.2.4 FTIR Analysis

The attenuated total reflection-Fourier transform infrared spectroscopy (ATR-FTIR; PerkinElmer, Waltham, USA) was used as a nondestructive method for detecting the functional groups and elemental compositions of the samples. The range between 4000 cm^–1^ and 400 cm^–1^ was chosen to measure 18 survey spectra for each sample, i.e., powdered compounds (LF, LB), solutions (PEO, PEO/LF, PEO/LB, PEO/LF/LB), and all the electrospun samples (ePP, ePEO, ePEO/LF, ePEO/LB, ePEO/LF/LB).

#### 2.2.5 Surface Chemical Composition Analysis: XPS

The quantitative surface chemical composition of the electrospun samples was analyzed using X-ray Photoelectron Spectroscopy (XPS) [PHI-TFA 5600 XPS (Physical Electronics inc., USA)]. The XPS spectrometer irradiated the sample with monochromatic Al Kα X-ray light with a photon excitation energy of 1486 eV. The characteristic peaks for the elements present on the sample surface to a depth of about 6 nm were recorded by the acquisition of survey and high-resolution spectra. An electron gun was used for surface charge neutralization during the measurements.

#### 2.2.6 Surface Morphological Analysis

The morphology of the electrospun samples was determined by an FE-SEM Supra VP 35 instrument (C. Zeiss AG, Germany). A dry sample was attached to an aluminum carrier by applying a conductive carbon strip. Palladium (Pd) was used for improving the conductivity. Prior to placing the sample in the apparatus, the samples were blown with nitrogen to prevent contamination. The applied voltage was 1 keV, while a variable working distance and 30 μm apertures were used.

#### 2.2.7 The ABTS Method

The ABTS (2,2′-azino-bis­(3-ethylbenzothiazoline-6-sulfonic acid)) method is considered an indirect method of determining the antioxidant potential, as it is based on the measurement of the antioxidant’s ability to scavenge free radicals that are not related directly to oxidative degradation. These ABTS^•+^ free radicals are formed by oxidation between ABTS and potassium persulfate (K_2_S_2_O_8_). The absorption maximum is at a wavelength of 734 nm. The green-blue-colored ABTS^•+^ is reduced in the presence of antioxidants, resulting in a change in the absorbance, which leads to the discoloration of the solution. Since the ABTS^•+^ radical is soluble in both aqueous and organic solvents, the method is useful for determining the antioxidant potential of hydrophilic and lipophilic antioxidants.^32^ The antioxidant potential was determined by initially weighing 0.1 g of the sample into a test tube and adding 3.9 mL of ABTS free radical to it. The radical inhibition was determined spectrophotometrically by measuring the absorbance at 734 nm and 25 °C at time intervals of 15, 30, 45, and 60 min. The antioxidant efficiency is expressed as a percentage value of the free radical inhibition (IEt), and it is calculated as the average value of three consecutive measurements according to eq 1:

$$IEt=\left(A_{\mathrm{initial}}-A_{\mathrm{final}}\right)/A_{\mathrm{initial}}\times 100\%$$ 1

where IEt is the inhibition of free radicals (%), *A*_initial_ is the measured absorbance at the initial ABTS^•+^ concentration, and *A*_final_ is the measured absorbance at the remaining concentration of ABTS^•^.

#### 2.2.8 Assessment of Lactobacilli Release and Survival

To determine the effect of storage conditions on the survival of LK7, each electrosprayed sample (1200 cm^2^) was divided into three equal parts of 400 cm^2^. One third of the sample was analyzed fresh within 90 min, while the remaining thirds were stored in a refrigerator (8 °C) and a climate chamber (20 °C and 65% relative humidity), and then, after 3 days of storage, the latter samples were subjected to a survival and release analysis.

The material (400 cm^2^) was placed in a sterile bag; 100 mL of PBS buffer was added, and everything was homogenized using a stomacher (BagMixer, France). At certain time intervals (1, 5, 10, 15, 20, 25, 30 min), 1 mL of the diluted samples was inoculated in Rogosa agar to determine the cfu/mL. The Petri plates were incubated under anaerobic conditions at 37 °C for 72 h. The anaerobic conditions were established using the Genbox anaer system (Biomerieux, Marcy-l’Étoile, France).

## 3 Results and Discussion

### 3.1 Electrospinning Parameters

Prior to electrospinning, all of the polymer formulations were characterized to determine their physicochemical properties, which are critical for fiber formation and process stability. Table 2 presents the measured pH, dynamic viscosity (η), electrical conductivity (κ), and surface tension (γ) for PEO alone and for composite formulations with lactoferrin (LF), *L. paragasseri K7* (LB), and their combination. These parameters influence the electrospinning jet behavior, fiber morphology, and the incorporation efficiency of bioactives.

**Table 2:** Formulation Characterization (PEO, PEO/LF, PEO/LB, PEO/LF, PEO/LF/LB) before Electrospinning

Solution	pH	η (mPas)	κ (μS/cm)	γ (mN/m)
s-PEO	7.91 ± 0.04	4009.7	100.4 ± 0.14	61.55 ± 1.93
s-PEO/LF	7.71 ± 0.03	12 197	257.23 ± 3.01	44.70 ± 3.023
s-PEO/LB	8.37 ± 0.16	2771.5	514.23 ± 2.80	57.83 ± 2.69
s-PEO/LF/LB	7.42 ± 0.04	4079.7	709.66 ± 2.81	52.20 ± 2.70

As expected, the addition of LF and/or LB altered the solution properties substantially. The PEO/LF solution showed a significant increase in viscosity (over 12 000 mPas) and conductivity with a concurrent decrease in surface tension. This is consistent with the known polyanionic and proteinaceous nature of LF. The LB-containing solutions exhibited markedly higher conductivity, particularly for the combined PEO/LF/LB formulation (709 μS/cm), due to the presence of bacterial cells, metabolic residues, and ionic components from the culture medium. These changes likely contributed to increased instability in the spinning jet and may have affected the bacterial incorporation efficiency, as reported previously in high-voltage electrospinning systems.^30^

The electrospinning process was optimized through systematic variation of both the technological and environmental parameters. The final settings were defined as 40 kV applied voltage, 210 mm electrode distance, and 3.8 rpm rotation speed of the lower roller electrode. The ambient conditions were kept at 21 ± 2 °C and 53 ± 5% relative humidity. Although this voltage was reduced by approximately one-third compared to previous studies, it remains relatively high, potentially causing partial damage to live bacteria or reducing their embedding efficiency into fibers. This may explain the surface-localized distribution of the lactobacilli observed by SEM and their immediate release during release studies. Despite these challenges, stable nanofiber formation was achieved across all of the formulations, highlighting the adaptability of the needleless electrospinning technique to complex bioactive-loaded systems.

### 3.2 Characterization

#### 3.2.1 The FTIR Spectra of the Formulations

s-PEO, s-LB, s-LF/LB, s-PEO/LF, s-PEO/LB, s-PEO/LF/LB, and LF in powdered form are seen in Figure 1. In the spinning formulation of PEO, the presence of two functional groups was observed: −CH (3300 cm^–1^) and C=O (1640 cm^–1^). In the LF powder sample, signals for −OH and C=O groups and a signal at 1390 cm^–1^ illustrating the presence of the −OH group of phenol were also detected at wavelengths of 3300 cm^–1^ and 1640 cm^–1^. At a wavelength of 1490–1580 cm^–1^, the −NH group was also noticeable. The spectra of the liquid formulations sPEO/LF, sPEO/LB, sPEO/LF/LB, and sLB coincided almost completely with the spectrum of the PEO formulation; therefore, two functional groups appeared −CH (3300 cm^–1^) and C=O (1640 cm^–1^). −OH (3290 cm^–1^), C=O (1640 cm^–1^) and −NH groups at a wavelength of 1350 cm^–1^ were observed in the LF/LB substance.

**Figure 1:**
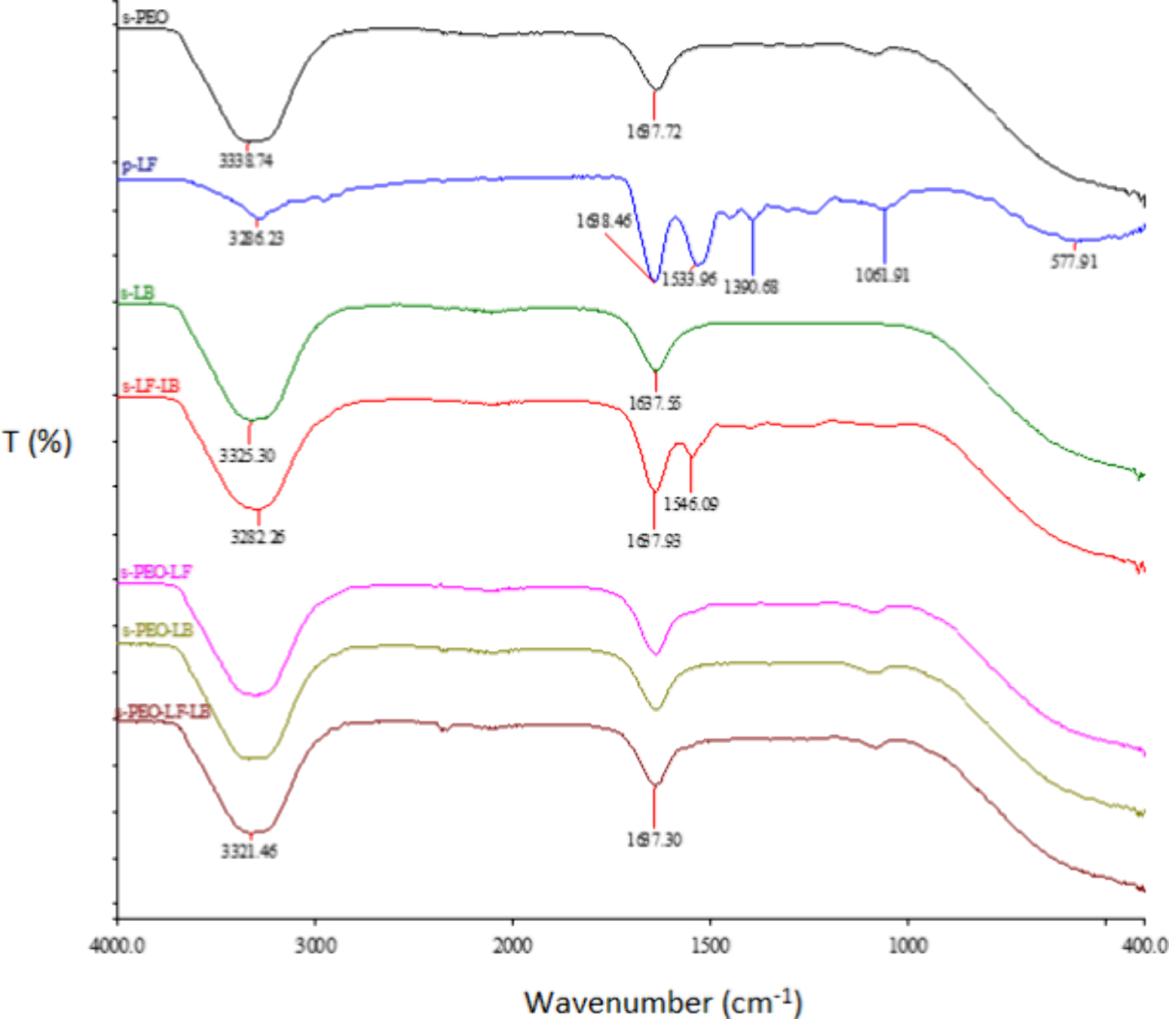
FTIR spectra of the formulations s-PEO, s-LB, s-LF/LB, s-PEO/LF, s-PEO/LB, and s-PEO/LF/LB and p-LF.

Figure 2 shows the FT-IR spectral peaks of the reference PP fabric and electrospun samples of PEO, PEO/LF, PEO/LB, and PEO/LF/LB. In the case of the reference carrier PP fabric, we detected a multiplet of signals at the wavelength of 2900 cm^–1^, which showed the presence of C–H bonds, and a signal at 1375 cm^–1^, characteristic of oscillation of the −CH_3_ bond. The spectrum of the electrospun PEO sample contained a signal between 2800 and 3000 cm^–1^, which represented the C–H functional group, and a C–OH group whose signal was located at a wavelength of 1000–1300 cm^–1^. It can be seen from the FTIR spectrum that the advanced PEO/LF, PEO/LB, and PEO/LF/LB samples had almost the same chemical structure as the PEO sample before. In the samples mentioned, the functional group C–H between 2800 cm^–1^ and 3000 cm^–1^ was recognized, further C–OH at 1000–1300 cm^–1^ and the −CH_2_ bond at 1455 cm^–1^.

**Figure 2:**
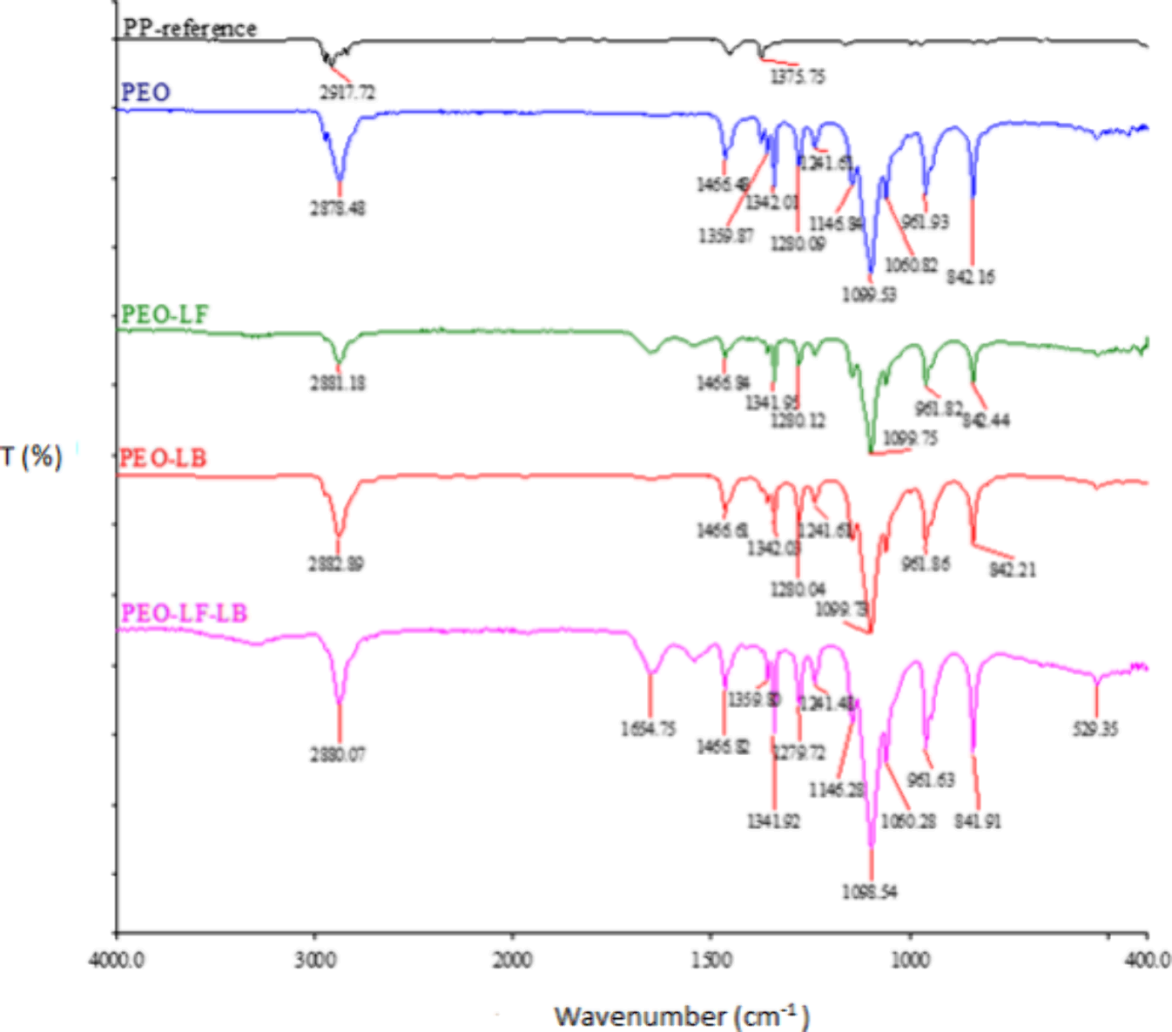
FTIR spectra of the PP and electrospun samples PEO, PEO/LF, PEO/LB, and PEO/LF/LB.

The ATR-FTIR analysis confirmed the successful incorporation of lactoferrin and *L. paragasseri* K7 into the PEO matrix, as evidenced by the characteristic functional groups −CH, −OH, C=O, and −NH. The spectra of the electrospun samples resembled that of pure PEO closely, indicating that, while bioactive components are present, their signals are partially masked by the dominant polymer structure. Complementary techniques like XPS were required to further verify the integration of functional additives.

#### 3.2.2 Surface Chemical Composition

The surface chemical composition of the electrospun samples was validated by X-ray photoelectron spectroscopy (XPS). Table 3 shows the results of the electrospun samples and the reference PP.

**Table 3:** Element Composition of PP and Samples PEO, PEO/LF, PEO/LB, and PEO/LF/LB

Sample	C (%)	N (%)	O (%)	P (%)	K (%)	Na (%)	Ca (%)
PP	68.9		20.5	4.1	5.6	0.9	
PEO	57.8		33.4	3.1	5.7		
PEO/LF	63.6	2.7	31.9	1.0	0.8		
PEO/LB	59.7		33.5	2.5	2.6	1.4	0.3
PEO/LF/LB	66.6	1.2	32.0	0.2			

Compared with the PP reference, C and P were present in all the samples in lower concentrations, while O was in the higher concentrations. It shows that the electrospun material prepared was efficient. Beside the enumerated elements, K was also found (except in PEO/LF/LB). The functionalized sample PEO/LF also contained N, while Ca and Na were found in sample PEO/LB, as is evident from Table 3.

The XPS analysis validated the successful surface functionalization of the electrospun fibers with biologically active components clearly. The observed nitrogen peak in the PEO/LF sample confirmed the presence of proteinaceous material—consistent with lactoferrin adsorption, as nitrogen is a distinctive marker of peptides and proteins.^33^ Meanwhile, the detection of calcium and sodium in the PEO/LB sample indicated the incorporation of bacterial components since these elements are characteristic of microbial cell surfaces and intracellular content. Moreover, the increased oxygen content across all the functionalized fibers compared to the PP control supported the successful binding of biologically relevant molecules to the nanofiber surface further.

#### 3.2.3 Surface Morphology Results

Figure 3 shows SEM microscopic images of the formed nanofibrous structure PP (as reference material) (a) and PEO (b), PEO/LF (c), PEO/LB (d), and PEO/LF/LB (e). A magnitude of 1000-fold was applied. The measure at 20 μm was denoted on the SEM micrographs.

**Figure 3:**
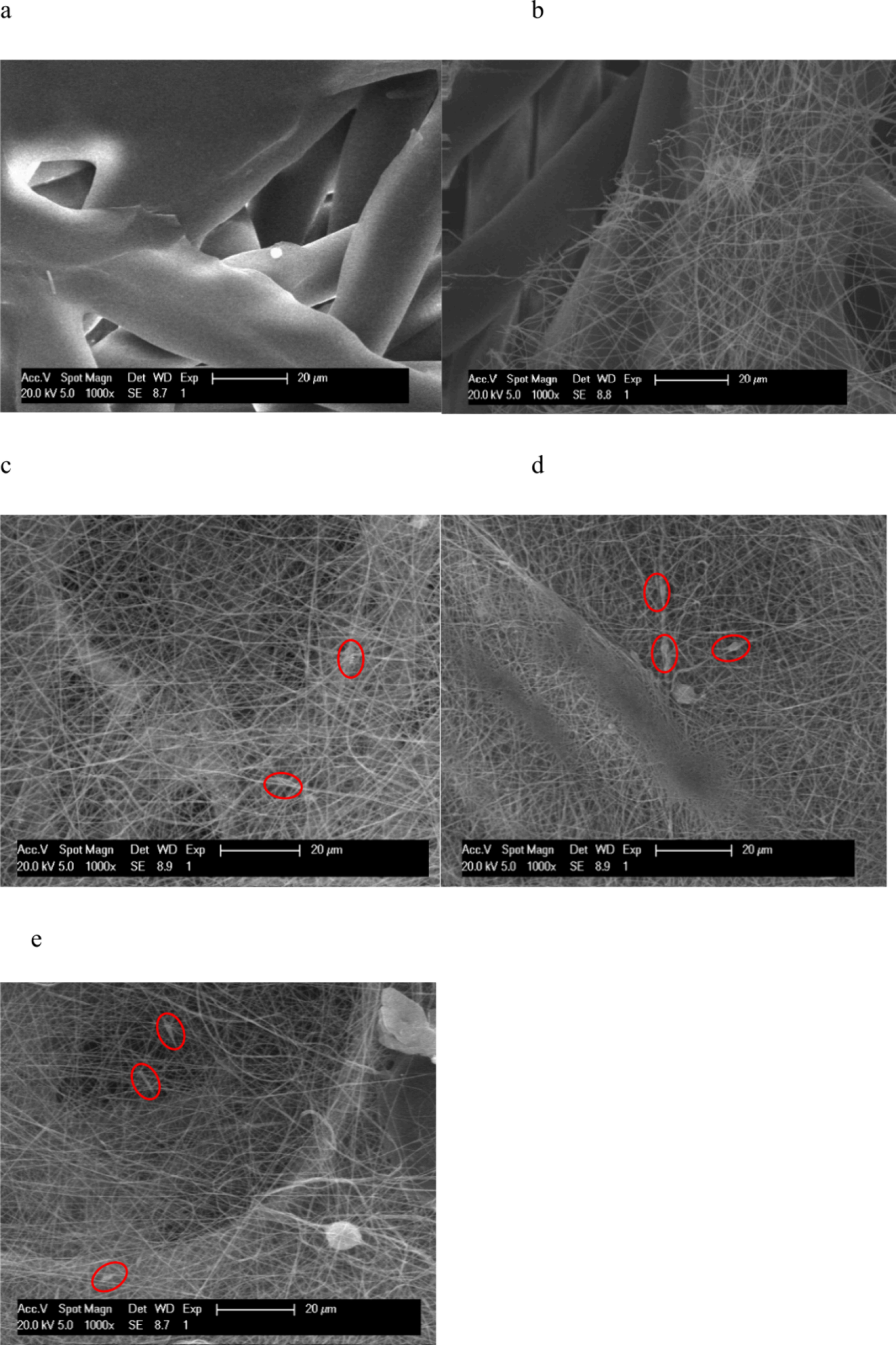
SEM micrographs: PP (a), PEO (b), PEO/LF (c), PEO/LB (d), and PEO/LF/LB (e).

Figure 3a represents the micrograph of the basic polypropylene matrix. The fiber diameter was approximately 22 μm. Macroscopic particles can also be observed, which may have been formed during the production of the PP fabric itself. The SEM image in Figure 3b (the electrospun PEO sample) shows that the thick propylene nanofibers were derived from the PP fabric, and a dense network of thin PEO nanofibers with a diameter of about 1.3 μm that were spun onto the PP is observed. It was found that the PEO nanofibers were also formed when Lf or/and lactobacilli were added to the formulation (Figure 3c–e). In accordance with another study, the SEM demonstrated that LF influences probiotic adhesion onto electrospun material.^34^ A film of the undissolved formulation was present.

Figure 3d shows the SEM image of the electrospun PEO/LB material: PEO nanofibers were developed, while the LK7 probiotics remained on the surface and were not incorporated into the resulting nanofibers. Bacterial localization within the electrospun fibers is governed not only by cell size but also by the fiber diameter and spinning solution rheology. However, the direction of the lactobacilli was linear along the fiber direction, which coincided with another study.^35^ The diameter of the resulting PEO nanofibers was around 1.3 μm. The lactobacilli were characterized by a length between 1 and 1.5 μm and a diameter of 0.7–1.0 μm.^30^ A comparable value to the diameter of PEO nanofibers with the diameter or length of lactobacilli can be an obstacle or limitation in incorporating LB into nanofibers. Moreover, the viscosity and conductivity of LB-containing formulations hindered encapsulation and aligned bacteria on the fiber surface. Such localization facilitates immediate bacterial release, which is consistent with the intended application. Comparable results were reported previously by Stojanov et al.^8^

The formation of polyethylene nanofibers was observed in Figure 3e (the PEO/LF/LB sample). These were thicker in individual places. Since the diameters of the nanofibers and lactobacilli coincided almost completely, they could not be incorporated into the PEO nanofibers effectively. The presence of two larger macroscopic particles was also pronounced because of the aggregation of the lactobacilli cells.^36^

To summarize, the SEM imaging confirmed the successful formation of PEO nanofibers on the polypropylene (PP) support with consistent fiber diameters of around 1.3 μm. The addition of lactoferrin and/or *L. paragasseri K7* did not hinder fiber formation. However, the probiotic cells were not embedded within the fibers; instead, they remained aligned along their surfaces—likely due to their size being comparable to the fiber’s diameter. In formulations with both LF and LB, occasional aggregation of the bacterial cells was observed, confirming the surface retention rather than encapsulation further. These findings highlight the need for optimized conditions to improve bacterial entrapment in future formulations. However, despite the lack of bacterial incorporation within the nanofiber core, the surface localization of *L. paragasseri* K7 enabled immediate contact with the target mucosa, making the electrospun mats functionally suitable as rapid-release delivery platforms.

### 3.3 Antioxidant Potential of the Electrospun Formulations

#### 3.3.1 Before Electrospinning

The antioxidant potential of the powdered LF (p-LF) and aqueous solutions (s-PEO, s-PEO/LF, s-PEO/LB, s-PEO/LF/LB, s-LB/LF, and s-LB) was determined before electrospinning. The results of the initial absorbance and those after 15, 30, 45, and 60 min are shown in Table 4.

**Table 4:** Absorbance of p-LF and Formulations after 15, 30, 45, and 60 min

Solution	*A*_initial_	*A*_15_	*A*_30_	*A*_45_	*A*_60_
s-PEO	0.7650	0.7264	0.7059	0.7026	0.4961
s-PEO/LF	0.7650	0.5742	0.4268	0.3307	0.2888
s-PEO/LB	0.7650	0.7634	0.7319	0.6608	0.6281
s-PEO/LF/LB	0.7650	0.5252	0.4266	0.4108	0.3846
s-LB/LF	0.7065	0	0	0	0
s-LB	0.7038	0	0	0	0
p-LF	0.7038	0	0	0	0

The more the absorbance decreased, the more the antioxidative activity increased. Table 4 shows that the absorbance decreased in all of the solutions during 60 min. However, the decrease of absorbance in the last three samples (s-LB/LF, s-LB, and p-LF) occurred in less than 15 min.

The inhibition of free radicals was calculated according to eq 1, and the results are shown in Figure 4. For LF, as well as for LB and LF/LB, there was 100% radical inhibition, meaning high antioxidant potential already after 15 min. It was decreased slightly by the addition of PEO.

**Figure 4:**
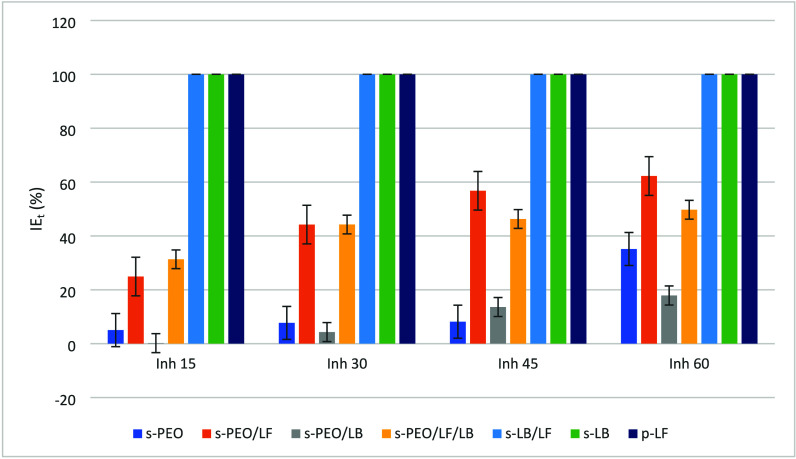
Inhibition of samples over time: sample p-LF and solutions s-PEO, s-PEO/LF, s-PEO/LB, s-PEO/LF/LB, s-LB/LF, and s-LB.

#### 3.3.2 Antioxidant Potential of the Electrospun Material

In the reference material PP and in the samples PEO, PEO/LF, PEO/LB, and PEOL/LF/LB, the absorbances were measured for the solutions. The results are shown in Table 5. The calculated inhibition (%) is also presented in Figure 5.

**Table 5:** Electrospun Material Absorbance Measurements

Sample	*A*_initial_	*A*_15_	*A*_30_	*A*_45_	*A*_60_
PP	0.7315	0.6609	0.6084	0.5968	0.5733
PEO	0.7010	0.6594	0.6221	0.6188	0.5603
PEO/LF	0.7010	0.5421	0.4998	0.417	0.3645
PEO/LB	0.7010	0.6537	0.6038	0.5573	0.5341
PEO/LF/LB	0.7010	0.5512	0.3689	0.2606	0.2187

**Figure 5:**
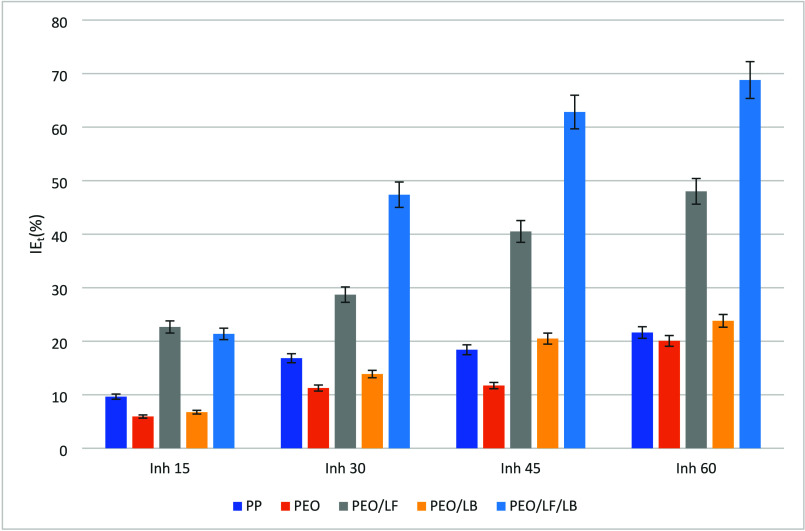
Inhibition of PP and electrospun material: PEO, PEO/LF, PEO/LB, and PEOL/LF/LB.

Before, the PP-fabric and PEO samples had the lowest level of free radical inhibition. After 20 min, the IEt of the mentioned samples did not exceed 20%, so we can say that, before, the PEO sample and the PP reference did not show antioxidant potential. The fibers with PEO/LF showed a slightly higher level of free radical inhibition than the PEO/LB fibers, and because the electrospun sample showed the highest effective antioxidant activity PEO/LF/LB, it can be concluded that the addition of LF increased the inhibition level. It could be attributed to the alteration of the LF structure and, consequently, increased the contactable area of LF.^37^ The values of the antioxidant potential of the electrospun nanofibers were lower than those of the individual substances, so it could be claimed with high probability that the electrospinning affected the reduction of the antioxidant effect of the nanofibers. This observation could be attributed to the inhibition of LB by PEO.^30^ The electrospun PEO/LF/LB fibers demonstrated the highest antioxidant activity among all of the tested samples, indicating that the synergistic effect of lactoferrin and *Lactobacillus paragasseri* K7 is preserved despite the electrospinning process. While high-voltage fabrication induced a minimal reduction in overall activity, the formulation retained biologically significant radical-scavenging potential. Lactoferrin’s antioxidant capabilities are well-established, particularly in mucosal environments—such as vaginal or amniotic fluids—where it reduces oxidative stress significantly by chelating iron and scavenging reactive oxygen species.^36^ The antioxidant role of lactobacilli has been well-documented across multiple studies. For example, *L. brevis* and *L. gasseri* showed over 90% DPPH radical scavenging activity in vitro. Additionally, *L. plantarum* strains have been reported to tolerate high concentrations of hydrogen peroxide and exhibit strong in vitro antioxidant capacities, including free radical scavenging and upregulation of antioxidant defense. A comprehensive review confirmed the ability of various *Lactobacillaceae* species to alleviate oxidative stress by scavenging reactive oxygen species, chelating metals, and enhancing host antioxidant enzyme activity.^38^ The antioxidant activity was comparable to another study with PEO-based nanofibers containing 5% gallic acid.^39^

Collectively, these findings support the hypothesis that the potent ABTS activity observed in the PEO/LF/LB formulation is due to the combined action of lactoferrin’s iron-chelating and ROS-neutralizing properties with antioxidative metabolites (like glutathione and exopolysaccharides) produced by *L. paragasseri* K7. This multifunctional antioxidant defense could be critical in reducing oxidative damage, inflammation, and epithelial stress in vaginal tissues, underlining the material’s promise for therapeutic applications in conditions like BV or mucosal injury.

The pronounced antioxidant activity observed in the PEO/LF/LB formulation is supported strongly by findings from complementary analytical techniques. The FTIR and XPS analyses confirmed the presence of functional groups and elemental markers associated with biologically active molecules—such as nitrogen- and oxygen-containing groups from the lactoferrin and bacterial structures—which are known contributors to redox activity. These chemical signatures suggest that the antioxidant function is not incidental but embedded within the material structurally.

The SEM imaging revealed that the *L. paragasseri* K7 cells were not encapsulated inside the fibers but adhered to their surfaces, a configuration supported further by the rapid release results showing immediate probiotic availability upon exposure to aqueous media. This rapid release is essential for prompt biological action, including free radical scavenging in oxidative environments such as inflamed or dysbiotic vaginal mucosa.^40^

These results indicate that the antioxidant activity arises from a synergistic combination of structural, chemical, and microbiological factors. The optimized fiber morphology, surface availability of both lactoferrin and probiotics, and their confirmed functional presence all contribute to the high radical-scavenging potential of the electrospun materials. This multimodal evidence highlights the formulation’s potential as a fast-acting, topically applied antioxidant system for vaginal or mucosal applications.

In addition to their antioxidant activity, both lactoferrin and *L. paragasseri* K7 demonstrated antimicrobial effects against a broad spectrum of bacteria, including species commonly associated with urinary tract infections such as *Escherichia coli*, *Staphylococcus aureus*, and *Enterococcus faecalis*.^27,31,41^ Moreover, bacteriocins produced by *L. paragasseri* K7 are effective against *Lactobacillus iners* and *Gardnerella vaginalis*, which are associated with bacterial vaginosis.^42^

### 3.4 *L. paragasseri* Survival and Release

The results of the analysis of viability of the strain LK7 from the electrospun PEO/LB and PEO/LF/LB samples are presented, namely: fresh, stored for 3 days in a refrigerator (8 °C), and stored for 3 days in an air-conditioned chamber (20 °C and 65% relative humidity). The release kinetics took place in 5 min intervals from the first to the 30th min. The results were given as the average of three measurements of the proportion of released bacteria after the time intervals (1, 5, 10, 15, 25, 30 min).

Figures 6 and 7 show the % of survived and released *L. paragasseri* K7 cells in the electrospun samples (enumerated as % cfu) in relation to the initial amount of lactobacilli introduced into the electrospinning formulation and calculated to the corresponding surface.

**Figure 6:**
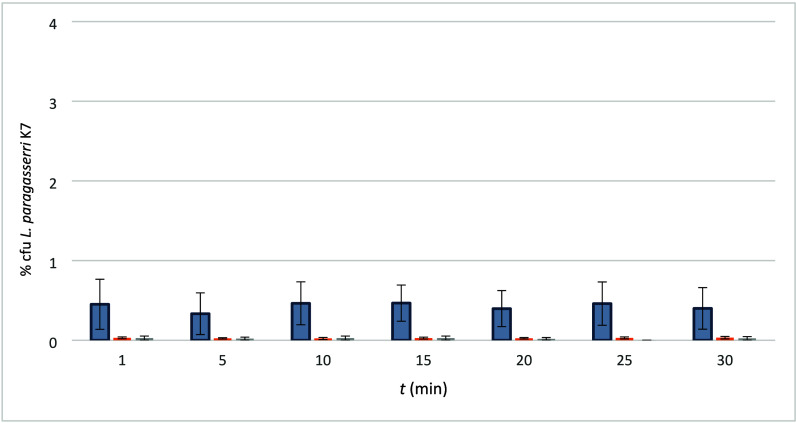
Ratio (%) of survived and released LK7 from the sample PEO/LB; gray = fresh, orange = fridge, light gray = airconditioned chamber. % cfu *L. paragasseri* K7 represents the ratio of LF cfu in comparison with the initial number of cfu in the electrospinning formulation, calculated at the same surface of the electrospun product.

**Figure 7:**
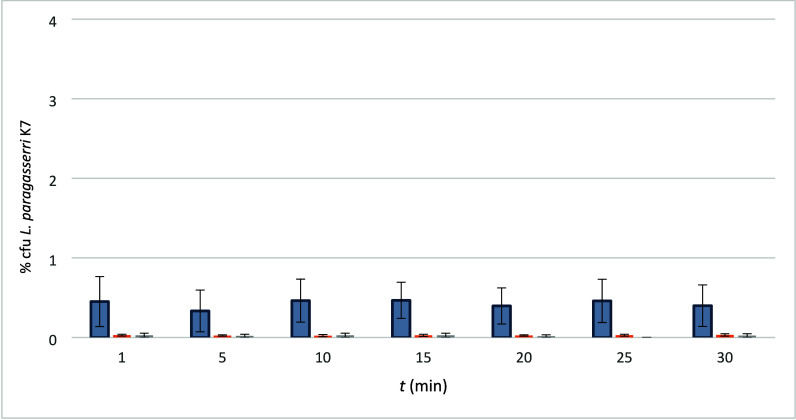
Ratio (%) of survived and released LK7 from PEO/LF/LB. Gray = fresh, orange = fridge, light gray = airconditioned chamber. % cfu *L. paragasseri* K7 represent the ratio of LF cfu in comparison with the initial number of cfu in the electrospinning formulation, calculated at the same surface of the electrospum product.

It is observed clearly that the % cfu of lactobacilli detected in the fresh samples immediately after placing the samples into PBS (fresh sample, 1 min) did not increase during 30 min of incubation in PBS, which indicates that all the bacteria were already released at this first sampling, in accordance with another study.^43^ The highest proportion of bacteria was determined in the fresh nanofibers, as the K7 viable count (cfu) was 0.5% of the initial CFU in suspension, calculated on the same fabric surface area. So, the result of the % cfu determination in the fresh fabric samples (1 min) shows that 0.45 ± 0.25% cfu (PEI/LB) or 0.38 ± 0.18% cfu (PEO/LF/LB) of lactobacilli survived the process of electrospinning. A similar decrease in viability after electrospinning has been reported by Ilomuanya et al. (3-log reduction in nanofibers)^44^ and Zupancic et al. (up to 3-log reduction in PEO fibers).^45^ Only viable lactobacilli capable of multiplying and forming colonies were detected by the plate counting method. We should consider, however, that more bacteria might have survived but were in a nonculturable state (VBNC, viable but nonculturable).^46^ A lower value of surviving LB was observed in the samples kept for 3 days in the refrigerator and in the climate chamber. However, since the survival rate depends on various process conditions, the growth phase, and several other factors, there are several possibilities for optimization that lead to a better survival rate of the lactobacilli.^47^ The addition of cryoprotectants, e.g., trehalose, was found to enhance the viability of probiotic cells in the composite nanofibers PEO:cryoprotectant significantly for longer periods.^48^

As mentioned above, a certain proportion of bacteria could have survived the storage process under the mentioned conditions but been in the VBNC state. Other methods, such as viability staining and microscopic enumeration or viable real-time PCR methods, should be used to support the interpretation of probiotic viability loss during electrospinning further, together with ROS-sensitive probes to detect oxidative damage, leakage assays to monitor the release of intracellular contents as indicators of envelope disruption, and microscopy techniques (SEM, TEM, fluorescence) to visualize the morphology and bacterial localization within or on the fiber surface. In line with previous studies,^8,10,49–52^ these approaches provide a robust framework for identifying process-specific stressors such as shear forces, solvent effects, dehydration, and electric field-induced injuries. Incorporating such assays will be a key focus of our future work to confirm mechanism-specific damage and guide optimization of the formulations.

Although the initial viability of *L. paragasseri* K7 was low, the observed antioxidant activity in the LF/K7 formulations and previously reported antimicrobial activity of both components^27,31,41^ suggest that the postbiotic components released from damaged cells, in combination with LF, contributed to the preserved bioactivity. This is consistent with the concept of postbiotics as health-promoting agents with antioxidant, antimicrobial, and immunomodulatory properties.^53,54^

Taken together, the results demonstrate that the electrospun PEO fibers allowed for the immediate and complete release of viable *L. paragasseri* K7 cells with release occurring entirely within the first minute of contact with the aqueous medium. The exceptionally rapid release, in combination with survival rates above 0.3%, highlights the functional potential of the material as a short-contact delivery system. Importantly, the lack of additional release after 1 min and the SEM evidence of surface-localized bacteria suggest a burst-release mechanism rather than diffusion-controlled release. While the survival rate may appear modest, the use of fresh biomass and the absence of cryoprotective excipients underline the system’s efficiency under unoptimized conditions. Future formulation strategies, such as polymer blending or modified electrospinning parameters, could enhance bacterial viability while preserving the rapid-release characteristics that make the material promising for vaginal or mucosal applications requiring immediate therapeutic action.

#### 3.4.1 Results of the LB Release from the Electrospun Material PEO/LF/LB

The results are presented in Figure 7.

Compared with PEO/LB, the release with PEO/LF/LB was generally comparable under all conditions according to Figures 6 and 7. The addition of LF did not improve the survival and release of LB. It was hypothesized that the viability of probiotics during storage depends on their moisture uptake characteristics.^12^ The electrospun material had a moisture content of 53 ± 5%. It was reported that, at 55% relative humidity, the electrospun nanofibers were the thinnest (diameter 81 ± 18 nm), resulting in a bead-like morphology that was not the consequence of incorporated *Lactiplantibacillus plantarum*.^48^ However, it was demonstrated that LB could survive at lower temperatures, but the release was not improved compared to the results obtained at room temperature.

In summary, the addition of lactoferrin did not enhance the viability or release efficiency of *L. paragasseri* K7 significantly compared with the PEO/LB formulation, suggesting that LF does not function as a stabilizing excipient under the tested electrospinning conditions. Nevertheless, the results from the complementary analyses offer critical insight: FTIR and XPS confirmed the presence of nitrogen- and oxygen-containing functional groups typical of proteins and bacterial cell components, supporting the successful incorporation of both lactobacilli and LF into the fiber–matrix. Furthermore, the SEM analysis showed surface-associated bacteria clearly, providing a structural explanation for the immediate release observed, where the viable cells were released fully within the first minute of contact with PBS. Despite the lack of structural stabilization benefits from LF, its copresence with *L. paragasseri* K7 resulted in the highest antioxidant activity across all the tested formulations, indicating preserved biofunctionality postprocessing. Altogether, these findings underline that the probiotic–lactoferrin synergy is likely functional rather than structural and that further formulation optimization could enhance bacterial stability while maintaining the rapid-release profile suited for vaginal and mucosal therapeutic applications.

Future improvements may include the use of polymer blends, cryoprotective excipients, or multilayer electrospinning to extend the shelf life, improve bacterial survival, and tailor the release kinetics for both acute and sustained delivery scenarios. Previous studies confirmed that probiotic stability depends strongly on the formulation, packaging, and storage environment.^55^

## 4 Conclusion

Electrospun nanofibers were fabricated successfully using poly­(ethylene oxide) (PEO) with lactoferrin (LF), the probiotic strain *Lactobacillus paragasseri* K7 (LB), and their combination (PEO/LF/LB). Nanofiber formation was achieved in all the formulations, and scanning electron microscopy confirmed that the lactobacilli remained surface-associated, enabling direct environmental exposure. The antioxidant assays showed that both LF and LB exerted radical-scavenging activity individually, which was reduced slightly by incorporation into the PEO matrix and exposure to the electrospinning voltage. Notably, the highest antioxidant activity was observed in the combined PEO/LF/LB formulation, indicating a preserved synergistic biofunctionality after processing. In terms of viability and release, the lactobacilli demonstrated immediate and complete release from both functionalized materials (PEO/LB and PEO/LF/LB) within the first minute of immersion in PBS, with no additional release observed during the subsequent 30 min incubation. Although survival was reduced during storage, likely due to stress conditions and moisture interactions, the bacterial viability remained measurable, and the postbiotic potential was retained. Given that *L. paragasseri* K7 produces well-characterized bacteriocins (gassericins K7A and K7B), the material may also be exploited in postbiotic applications where bacterial viability is not required. Importantly, this study highlights the potential of nozzle-free electrospinning as a versatile platform for developing short-term, topical delivery systems for live biotherapeutics or postbiotics. While the survival rate of fresh probiotics was modest, the electrospun system offers unique advantages—including rapid release, high surface accessibility, room temperature fabrication without heat, and compatibility with sensitive bioactives. These characteristics are particularly valuable for mucosal or vaginal applications where immediate therapeutic action is desired. Future work should focus on optimizing the electrospinning process (e.g., to use core–sheath structures) to improve viability and shelf life of encapsulated biological components. Overall, the developed materials demonstrated promising applicability for medical textiles in women’s health—such as tampons or wound dressings—that support microbiota balance and mucosal recovery.
